# Valedictory Address to the Graduating Class of Rush Medical College

**Published:** 1854-03

**Authors:** J. V. Z. Blaney

**Affiliations:** Professor of Chemistry


### *Valedictory Address to the Graduating Class in Rush Medical College.*

By J. V. Z. Blaney, A. M., M. D., Professor of Chemistry.

Gentlemen of the Graduating Class—

You have, each of you, this evening reached a point in your own history which has doubtless been anticipated with alternating hopes and fears, as a goal to be attained, or as a rubicon to pass.

You are about to bid farewell to your preceptors and your associates, and henceforth dependent upon your own energies, to struggle for a position in a profession, whose ranks are crowded, and which numbers among its members men of age, talent, learning and experience. With few exceptions, you yourselves are wanting in three, at least, of those valuable qualifications, which others possess with whom you have to cope. It is presumed that you are youthful, while many of your professional brethren are aged; talent, indeed, you may have; a thorough knowledge of your profession you cannot be expected in the few years of your novitiate to have attained; and experience but to a limited extent can scarcely be yours. Under such circumstances it is not only customary and proper for us to offer, but you have a right to expect, from those who have been your preceptors during your course of study, in addition to a cordial farewell, some words of encouragement, of caution, and of advice. This duty I have been delegated by my colleagues to perform, and I could wish my ability were equal to my desire, to anticipate the exigencies of your future lives, and send you forth, armed at all points, to struggle and to conquer.

I will endeavor, as far as possible, in the short space within which I must limit my remarks, to present to you the most prominent difficulties which you may expect to encounter in your struggle for excellence in your profession, and the means by which they are to be overcome.

And first, I would remark that you are to *look within yourselves for the first and greatest obstacles which will obstruct your path to future eminence.* Know thyself is wisely held to be the summit of human knowledge. Could every one see as clearly the enemies he carries within his own bosom, as those which he encounters in the world, more than half the sources of human deficiency would be removed.

> "Oh! that the Lord the gift would gie us,
> To see ourselves as others see us,
> It would frae mony a trouble free us,
> An' foolish notion——"

I do not now refer to those more sordid and gross vices which beset all young men, thrown upon the cold charities of the world and their own exertions for their support, but the more subtle, and hence, more dangerous antagonists, indolence, diffidence, despondency, and indifference to the claims of the world and the profession upon their time, their talents, and their exertions.

Every man has more or less to strive against his own native indolence, but no class of men, in commencing the world, have as great temptation to be indolent as professional men, and among them none so great as the medical man.

The merchant and the mechanic find in their active duties something exciting and stimulating. The clergy have their weekly duties which must be performed. The lawyer has his term time and his justice courts to relieve the monotony of his office work. But the young physician has only his dull office and his books. In that dull office he must stay and wait the few calls with which his patience will, after a time, be rewarded. The long and tedious intervals between those few first calls must be spent with only gloomy reflections, and despondent forebodings as companions of his solitude. Days and weeks perhaps roll on, after he has opened his office, before the first call comes. Much depends upon that first call. What hopes and fears, self-confidence and distrust, what satisfaction that he now has the wished for opportunity to test his skill, and dread of assuming the responsibility of human life crowd each other through the mind of the young physician as he goes to pay his first call! He enters the house, takes his place for the first time by the bedside, unaided by his preceptor, and, for the first time, assumes the sole responsibility of a human life. If he be a bold man, his self-possession may carry him through successfully; but if he be a timid and a conscientious man, he will be overwhelmed with the responsibility, and perhaps scarcely retain sufficient self-control to examine the case so as to form, in his own mind, any decision upon it. If he were only a lawyer, he could tell his client to call to morrow, and meanwhile he would have time to examine his authorities, and perhaps consult his preceptor, and prepare a learned opinion, and that would be only a matter of dollars and cents. But alas! here there is no time to wait, he cannot go home to consult his books, for that would be to confess ignorance and abandon the case, and yet a human life is at stake. In this conflict regrets for time misspent in which the knowledge, now required, might have been gained, rises up in judgment against him and almost drives him mad. He hesitatingly prescribes something that he knows, or hopes, will not injure his patient, and hurries home to his dear old books, now prized as mines of gold, which yesterday were miserable old musty volumes not worth his notice. Now, indeed, he studies, if never before, and before he rises from his investigation he has possessed himself of all his small library contains that has any bearing upon the case in hand; and of this severe study the patient has the benefit at the next visit.

But how is it with the bold man, who has braved it through, and self-reliant, and self-possessed, succeeded in concealing from others, perhaps from himself, his own deficiencies? If he is a conscientious man, he also repairs to his office and consults his authorities; but if he is careless with his first case, he is hopelessly lost to himself and his profession. Indolence and overweening self-esteem have him in control and will lure him to his ruin. Young gentlemen, if you do not feel the responsibility of your profession pressing upon you in the earlier part of your professional career, before experience has given you confidence in your judgment and your remedies, you had better leave your profession and try something else.

As I have already said, much depends upon your first case. If your treatment proves successful, and your patient is restored to full and glowing health, the profession will be viewed by you in its most favorable aspect, and everything will wear the color of the

But should you lose your first case, and should you be a man of feeling, it will be long before the pale cheek, the lack-lustre eye, the stiffened limbs, will cease to haunt your sleeping and your waking hours. And what are the reflections which such an experience should excite? They are these:—To what cause is the death of my patient to be attributed? Is it to my indolence and consequent ignorance? This may be redeemed by future study, close application, unremitting diligence. Is it to any error in treatment or mistaken diagnosis? This may be due not to my want of application, but to my want of experience, or to the deficiencies of the science itself. If to either I am scarcely responsible for the result, as I have yet to make my experience; and I am not to blame for the deficiencies of the science up to this time. But if it be the latter, the class of cases to which it belongs requires investigation. I will undertake that investigation and redeem a life by giving to the profession the required means of saving many others. Such a determination carried out with energy and perseverance will relieve your waking hours of the load of a life, and banish the phantom from your dreams.

These should be your reflections and your resolves under the circumstances presented. But too often, disgust for the profession and a loss of confidence in its teachings, and in his own ability to ever attain any excellence in its ranks, is the consequence of an unfortunate occurrence to a sensitive man in the earlier years of his experience.

Do you wish to avoid those sleepless nights, those harassing thoughts which never fail to follow as legitimate consequences of duties unperformed, and of responsibility assumed for which you are unprepared, let me urge upon you to devote the intervals of active duty in the earlier years of your practice, in storing your minds with accurate information from all possible sources within your reach. The leisure you may then command you will never find in after life. When the ceaseless round of professional duties come upon you, you will look back with serious regret to those hours of your early history which were spent in frivolous amusements, listless idleness, or unavailing despondency.

The next point which I would urge upon your notice is, *the importance of keeping up with the constant advance of your profession.* But too often is it the case that the physician, who, during his term of study was a diligent student, and left his Alma-Mater glowing with zeal and professional pride, becomes, after a few years, a mere work-horse, performing day after day his usual routine of professional calls, and, satisfied with the pecuniary returns of his labor, settles down into a condition of perfect apathy, so far as ambition to excel or desire to add to the sum of professional knowledge is concerned. This may and frequently does occur to practitioners in cities and towns of considerable size, though from the constant attrition against other and numerous rival claimants for public confidence, the necessity of keeping pace with the march of medical science is an absolute essential to maintaining even a respectable position. Such however is not the case with physicians in villages or isolated districts. Dividing the business of the neighborhood with one or more professional brethren, and each having his own appointed ride, with none to disturb or arouse him from his professional sleep, he lives on from year to year, firmly believed by his patients to be a model Esculapius, and himself half persuaded to the same opinion, neither dreaming nor caring for any advance the profession may have made, until perhaps some stripling, fresh from the schools and well posted in everything which has been developed while he was enjoying his Rip Van Winkle sleep, suddenly appears as a rival in the field, and spite of all efforts to retain his position, strips him before he is well aware, of the life annuity he had fondly believed to be securely invested in the ignorance of his patients.

The means to be adopted to remedy any possibility of such lamentable deficiency are simple and obvious.

First to take, and diligently peruse one or more of the leading medical periodicals of the age. I would suggest to you to be in regular receipt of that journal, which best informs you of the general progress of the various departments of Medical Science, and also of the one which best represents the local interest of the profession in the region in which you may reside. The former will serve as a connecting link between you and the profession at large, the latter as a medium of communication between yourself and your professional neighbors. By the one you will be kept informed of every thing new and valuable which may be developed in the general field from time to time; by the other you will be stimulated by the example of the most energetic and best informed of your compeers to contribute your own share to the medical experience of your district.

You will thus be led to observe carefully all cases occuring in your own practice, and to institute such comparisons that while they develop the resources of your own mind may lead to results important alike to your own reputation and to the interests of humanity.

To attain these desirable results you must, however, subject yourself to a rigid and systematic course of discipline, closely to observe and invariably to record every case under your charge, which may in itself be unusual, or interesting, or which may tend to con firm, or disprove opinions or theories advanced by yourself or others. All matters of general interest to science should be carefully observed and noted, and whenever any series of observations shall have been completed, and the bearing of all the facts wel- compared and digested, it becomes your imperative duty to give to the world through the medium of a respectable periodical the results of your observations, experience and deductions.

By such a course of discipline the capabilities of your mind will be enlarged, your perceptions quickened, your interest in the profession kept alive, and you will be cheered and stimulated by the pleasing consiousness, that your sphere of usefulness and influence is extended beyond the narrow precincts of the social circle in which you reside.

Should you aim at more than mediocrity in the profession, let me recommend to you to *select as early as possible some one department of Medical Science*, to which your tastes or your locality may direct you, *and devote yourself to its study and advancement*. The science of medicine covers such an immense field that the duration one life is too short, and the opportunities of one man too limited, to appropriate all the knowledge which the labors of scientific men for centuries have accumulated. And, indeed, supposing any one man to have made his own, all the medical science of past ages, the advance of his own age is too much for his powers of application, especially when tramelled by the attention he is obliged to bestow upon the active duties of professional life.

The most then that any individual can expect, is to be able to become profoundly learned in some one department, and while making his professional reading sufficiently general to appreciate the advance in all, to be fully posted up, and have perfectly at his command the facts and details of one. Superficiality is the bane of science, and though it is asserted by some that he knows most who knows something of the greatest number of things, it is nevertheless also true that all great discoveries, and all eras in science have resulted from the concentration of the powers of scientific men to one single purpose and profound devotion to one single department of science. More particular attention to one department of science, does not however imply ignorance on inattention to others, but rather forces one. from mutual connections and in search of facts, to attain a certain amount of proficiency in others related to it. This special attention to one branch, moreover, has the effect to systematize ones study, gives it an aim and object, and forms habits of reflection and analysis. A man who reads superficially on many subjects to acquire other men's ideas, may become a walking cyclopedia of learning, but is not apt to become an original thinker. But let him severely confine himself to the perfect comprehension and rigid analysis of any one subject, and he soon ceases to take other men's dicta as truths, and begins to think for himself. He has then made one step toward that independence of thought, which properly directed may lead to the conception of new and great ideas. I would not for a moment undervalue general erudition, a man of studious habits employs his hours of relaxation from real absorbing study, in storing up general information, which will aid in his one great purpose. But it does not as has been asserted tend to contract one's intellect, to devote oneself to one great and special object. It may perhaps so appear to those who cannot appreciate one's aims or objects, just as it may be possible that a man whose fame is world-wide may be a person of but little consideration in his own village circle.

It is perfectly practicable for a practising physician, if he is well grounded in the rudiments and general literature of the profession in early life, to keep himself well informed in regard to new discoveries in all departments of the profession and still persue more profoundly one particular branch. The early selection of a favourite branch has other advantages. It guides one in the formation of his library, which in isolated districts is important from the difficulty of access to work of reference. It throws him into correspondence with members of the profession devoted to the same subject and he reaps the advantage of their advice, their criticisms on his own views, and the stimulus of their sympathy. In proof of the importance of the course I am recommending, look at the names of those most eminent in the profession for originality of research, at home and abroad, and you will find them all men specially devoted to one subject, it may be physiology, pathology, surgery, minute anatomy, or some other. Such are the men who make the progress of medical science, while the men of general reading without specific object assume the lower station of compilers, book makers, and journalists.

Again I say that I would not depreciate the value of general reading. If any man *should* be particularly well read in all departments of natural and mental science, that man is the physician. He has to deal, in his professional studies and duties, not only with the phenomena of matter, but of mind also. The influence of the mind in producing and modifying disease, in favoring or impeding the action of remedies, is so great, that the knowledge or ignorance of mental science, and of human passions and feelings will often make a wide difference in the success of practice of two physicians, in other respects, equally qualified. A thoroughly educated physician should then be a man of universal attainments. These attainments are to be considered a part of his primary education, and if not early acquired will be felt to be a deficiency until supplied. If however the groundwork be laid in early life, the full development will constantly progress, by an amount of study in after-life, which will not be viewed as laborious, but as giving variety, and hence relaxation, to the pursuit of more special subjects.

Another suggestion I would make—*to loose no opportunity of enjoying the intercourse of your professional brethren; especially when convened for mutual improvement in County, State, or National Societies.* Such meetings are not alone useful in deciding matters of professional etiquette, and promoting the welfare of the profession at large, but mainly, as I conceive, in stimulating individuals to increased effort, by contact with others equally or better informed than themselves, and exciting professional zeal, and the love of science *for itself*, and disconnected from pecuniary interests. In conventions of this kind, every man soon finds his own level. Those less informed finding themselves behind others, are stimulated to supply their deficiencies, and indeed may learn, for the first time, what their deficiencies are. This knowledge is often difficult to attain except by comparison with others, but when attained is the first step toward reformation.

Those meetings also recall to each individual matters in his own experience, which may have little prominence in his own mind for want of their proper bearings or relations. The remarks of another will occasionally throw new light upon facts of his own observation, and supply the connecting links which may lead to important deductions. Add to this the pleasures of professional intercourse, and the enlargement of one's circle of professional acquaintances, and the importance of this suggestion will be perceived.

*Fix your standard of professional excellence high, and aim to attain station equal to the highest.* Many men of excellent capacity have attained but mediocrity by fixing their standard of acquirement too low; many by an unfortunate location, which has brought them into contact with men, their inferiors in capacity, and of but medium acquirements, have satisfied themselves with distancing with ease their inferior competitors, and satisfied with their local reputation, have ceased from future exertion, and thus the world has lost the higher results it was within their power to have achieved. Let it not be so with you! Do not cramp your aspirations for high attainment by comparison with those around you merely, but compare yourselves with those who have done the most and the best for the profession, and cease not to labor till they acknowledge you as their compeer! You may, indeed, fail in your highest aim, but you will at least win higher than if your aspirations were lower. Be not daunted with difficulties! Despondency and the dread of failure belong to weak minds. "In youth's vocabulary there is no such word as *fail.*" But at the same time be not presumptuous! The disposition to overvalue one's self, is not the result of high aspirations or of comparison with one's superiors. If at any time you begin to feel superior to your professional associates, stop and ask yourself if you have no superiors, and then, in the consciousness of your own inferiority to others, be incited to nobler thoughts and feelings.

I have thus endeavored, gentlemen, to lay before you in a brief outline some of the principles which should guide you in pursuance of the duties devolving upon you by the assumption of the responsibilities of the medical profession. I might have extended my remarks much further and been more specific, but my parting words to you have been only intended to be suggestive of the demands of the profession and the world upon your talents and exertions, rather than of the special duties of individual practice. Of this duty one of my colleagues has relieved me by an address to you some days since, in which the relations of physicians to their brethren and their patients have been explicitly laid before you, and the moral obligations of your position in community defined.

It only remains for me on behalf of my colleagues and myself to bid you an affectionate farewell, and remind you that in closing our relations as preceptors and pupils, our interest in you, and your responsibility to us does not cease. You leave us to enter upon the duties of a profession which, next to Deity, holds the keys of life and death. It is for you to show, by your future success or failure in its arduous practice, whether or not we have performed our part towards you, and you have profited by our instructions. If we have failed in our duty to you, the responsibility of your acts rests with us; but if you are conscious of not having improved, to the fullest extent, the opportunities we have afforded you of a sound medical education, the moral load lie upon your own shoulders, and will only be relieved by persevering endeavors to redeem the time lost by your own neglect.

Nor is this all. You, each of you, go forth certified by us as prepared for the practice of the medical profession, and for the use made of that certificate, we must hold you responsible. Are you conscious of deficiencies, which you have succeeded in concealing, from us? we demand that you repair them to the utmost of your abilities and opportunities, that we, your instructors, and the Institution which acknowledges you, may not be charged with committing a fraud upon the public, or stand sponsors for the health and lives of patients, lost through your neglect. But while conjuring you thus, gentlemen, we confidently hope better things of each and all of you. For the past ten years the Institution which endorses you has depended for its reputation on the success of its graduates, and thus far, classes increasing in numbers and intelligence have, by seeking our halls for instruction, borne witness annually of the confidence of the public. With you, gentlemen, it remains, in a measure, to show whether or not this confidence shall be diminished or strengthened, and we hopefully trust that you will not disappoint our expectations. Remember, too, that you have a life-right in the course of instruction in your Alma Mater, and as the facilities of communication increase, and our means of teaching improve, by the constant additions we propose to make to our library, cabinets and museum, and continued endeavors to equal the best schools of our country, we expect you to embrace the opportunity afforded you, by occasional return, to reap the benefits of all improvements we may make. And when we, gentlemen, who have been your ushers to the threshold of medical science, shall have finished our work, may there be found among you those who will be ready prepared to fill our vacant places, and do more and better for the science and humanity than we have done.

Once more farewell, and God speed you in your work!

